# Evaluation of chitosan-GP hydrogel biocompatibility in osteochondral defects: an experimental approach

**DOI:** 10.1186/s12917-014-0197-4

**Published:** 2014-08-27

**Authors:** Edivaldo AN Martins, Yara M Michelacci, Raquel YA Baccarin, Bruno Cogliati, Luis CLC Silva

**Affiliations:** 1 Departamento de Cirurgia, Faculdade de Medicina Veterinária e Zootecnia, USP, São Paulo, SP, Brazil; 2 Departamento de Bioquímica, Escola Paulista de Medicina, UNIFESP, Rua Três de Maio, 100, São Paulo, 04044-020, SP, Brazil; 3 Departamento de Clínica Médica, Faculdade de Medicina Veterinária e Zootecnia, USP, São Paulo, SP, Brazil; 4 Departamento de Patologia, Faculdade de Medicina Veterinária e Zootecnia, USP, São Paulo, SP, Brazil

**Keywords:** Chitosan-GP, Cartilage, Proteoglycan, Type II collagen, Equine joint, Biocompatibility, Scaffold

## Abstract

**Background:**

Articular cartilage, because of its avascular nature, has little capacity for spontaneous healing, and tissue engineering approaches, employing different biomaterials and cells, are under development. Among the investigated biomaterials are the chitosan-based hydrogels. Although thoroughly studied in other mammalian species, studies are scarce in equines. So, the aim of the present study was to investigate the biocompatibility of chitosan-GP in horse joints submitted to high mechanical loads.

**Results:**

An osteochondral defect was created by arthroscopy in the medial surface of lateral trochlea of talus of left or right leg, randomly selected, from six healthy geldings. The defect was filled up with chitosan-GP. The contralateral joint received an identical defect with no implant. The chondral fragment removed to produce the defect was collected, processed and used as the “Initial” sample (normal cartilage) for histology, immunohistochemistry, and metabolic labelling of PGs. After 180 days, the repair tissues were collected, and also analyzed. At the end of the experiment (180 days after lesion), the total number of cells per field in repair tissues was equal to control, and macrophages and polymorphonuclear cells were not detected, suggesting that no significant inflammation was present. These cells were able to synthesize type II collagen and proteoglycans (PGs). Nevertheless, the cell population in these tissues, both in presence of chitosan-GP and in untreated controls, were heterogeneous, with a lower proportion of type II collagen-positives cells and some with a fibroblastic aspect. Moreover, the PGs synthesized in repair tissues formed in presence or absence of chitosan-GP were similar to those of normal cartilage. However, the chitosan-GP treated tissue had an disorganized appearance, and blood vessels were present.

**Conclusions:**

Implanted chitosan-GP did not evoke an important inflammatory reaction, and permitted cell growth. These cells were able to synthesize type II collagen and PGs similar to those synthesized in normal cartilage and in healing tissue without implant, indicating its chondrocyte nature.

## Background

Injuries to articular cartilage are common, and may cause pain, disability, impairment, and early retirement of athletes. Once damaged, articular cartilage has very little capacity for spontaneous healing, and many repair techniques have been proposed over the last decades. However, repair of articular cartilage by traditional methods is challenging, due to its avascular nature and lack of a significant population of progenitor cells. For this reason, tissue engineering approaches, employing cells to *in situ* regenerate articular cartilage, are under development [1]-[3].

Both fully differentiated chondrocytes and progenitor cells (mesenchymal stem cells) have been used to produce hyaline-like cartilage [4],[5]. Nevertheless, the strategies of cell implantation within the joint also poses challenges: a fluid environment, strong shear forces, mechanical loads, and stiff, irregular and often bleeding surfaces not easily amenable to insertion of biomaterials. Furthermore, the biomaterials to be used as cell-delivery vehicles must meet stringent requirements in that the material should be non-toxic, non-immunogenic, and must either integrate with the repair tissue or degrade without generating toxic by-products or leaving gaps, debris or fissures in the tissue [6]. The ideal scaffold should mimic the naturally occurring environment in the articular cartilage extracellular matrix (ECM), allowing chondrocyte proliferation and synthesis of cartilage ECM, which is composed by a dense network of collagen fibers [7], proteoglycans (PGs), and other proteins [8].

PGs are composed by a protein core with glycosaminoglycan (GAG) side chains. GAGs, in turn, are heteropolysaccharides constituted by repeating disaccharide units formed by a hexosamine (glucosamine or galactosamine) and a non-nitrogen monosaccharide (uronic acid or galactose). Most GAGs are sulfated in different degrees and positions, and both the carboxyl and the sulfate groups contribute to its polyanionic nature. The main cartilage matrix PG is aggrecan, a high molecular weight PG of the hyalectan family, composed by a ~200 kDa core protein substituted with ~100 chondroitin sulfate and ~30 keratan sulfate side chains. Aggrecan interacts with hyaluronic acid, forming large aggregates, which are entrapped in the collagen network, and provide cartilage its osmotic properties, giving cartilage its ability to resist compressive loads review in [9]. Other PGs, such as decorin, biglycan and fibromodulin, which are characterized by their ability to interact with collagen, are also present. They are much smaller than aggrecan, but may be present in similar molar amounts.

The structure and composition of aggrecan in articular cartilage changes when growth and calcification processes occur in the tissue, both during normal development and in diseases such as osteoarthritis and tumors [10],[11]. Furthermore, the extracellular matrix composition, the cell density [12], and even mechanical compression [13] affect the chondrocyte phenotype and the synthesis of ECM components. Thus, the composition of any biomaterial used as in cartilage regeneration procedures is very important.

A wide array of matrices or hydrogels have been used as biomaterials, including proteins (collagen and fibrin), polysaccharides (agarose, alginate, hyaluronic acid and chitosan), and polymers (polyethylene glycol, poly-lactic acid) [7]. Chitosan-based hydrogels are promising due to their structural analogy to GAGs.

Chitosan is a partially deacetylated derivative of chitin, isolated from arthropod exoskeleton [14]. It is a linear polysaccharide consisting of β (1–4) linked D-glucosamine residues, with a variable number of randomly located N-acetyl-glucosamine residues. Chitosan is a semi-crystalline polymer, and the degree of crystallinity is a function of the deacetylation level. Because of its structure, chitosan is normally insoluble in aqueous solutions above pH 7, but, in dilute acids, the free amino groups are protonated and the molecule becomes fully soluble below pH 5 [15].

Many chemical derivatizations of chitosan have been used to promote new biological activities and to modify its mechanical properties [16],[17]. The addition of polyol salts such as sodium glycerol phosphate to chitosan creates chitosan-glycerol phosphate (GP), a thermosensitive hydrogel that forms a viscous liquid at room temperature or below, and converts to a semisolid gel at body temperature [18]-[20]. Chitosan-based vehicle can be loaded into an osteochondral defect in living joints as a viscous liquid, and upon solidification, adheres to bone and to cartilage [15]. Furthermore, previous studies have shown that chitosan is hemostatic [21], and stimulates revascularization of the wound and connective tissue repair [22].

The aim of the present study was to investigate the biocompatibility of a chitosan-based hydrogel – chitosan-GP – in horses, specifically in joints submitted to high mechanical load. If proved biocompatible in horses, chitosan-GP could be used, in the future, as a vehicle for cell and/or drug delivery as an adjuvant to promote cartilage regeneration.

## Methods

### Animals

The present work was approved by the *Ethical Committee of Universidade de São Paulo – USP* (1245/2007), and was carried out in accordance with *USP guidelines*, and also in accordance with EC Directive 86/609/EEC for animal experiments http://ec.europa.eu/environment/chemicals/lab_animals/legislation_en.htm.

Six Mangalarga healthy geldings, 3 years old, 300–315 kg body weight (average = 307 kg), from the USP research herd, were used in the present study. Clinical, laboratorial, radiographic and ultrassonographic exams were performed before inclusion in the experimental group, and all were considered normal. The animals were housed in single 12 m^2^ boxes (3 × 4 m) and fed pellets (1% of the animal body weight), coast cross hay and water *ad libitum*.

### Preparation and application of chitosan-glycerol phosphate gel

Chitosan-GP gel was obtained as described by Chenite et al. [18]. In brief, chitosan^a^ of 310–375 kDa (based on the viscosity range of 800–2000 mPaS) deacetylation grade >75% (200 mg), was solubilised in 0.1 M hydrochloric acid (9 mL). To this solution, 560 mg of sodium glycerol phosphate^b^ dissolved in 1 mL of water were added, slowly and under continuous agitation. A whitish gel resulted, which was transferred to glass tubes, sterilized in autoclave, and stored for no more than 24 h at room temperature.

To test the biocompatibility of chitosan-GP, an osteochondral defect (1 cm diameter and 0.5 cm deep) was created by arthroscopy in the medial surface of lateral trochlea of talus, in left or right leg, randomly selected. A cartilage sample was collected with Ferris-Smith rongeous, and used as “Initial sample”. Afterwards, a depression was created in the subjacent bone using a bone burr attached to a power shaving system. This osteochondral defect was filled up with chitosan-GP gel (~0.5 mL). The gel was delivered using an insulin syringe (1 mL), with cut end. For gel delivery, fluid infusion was replaced by CO_2_, using a laparoscopic insuflattor calibrated to maintain 30 mmHg until the end of the procedure, allowing gel retention in the defect. The contralateral joint received an identical defect with no implant, and served as matched control.

After arthroscopy, all horses received intravenous amikacin, 15 mg/kg, and phenylbutazone, 4.4 mg/kg, every 24 h, for three days. At the end of the experimental period (day 180), arthroscopy was repeated for the collection of the “Final” samples, and the same antibiotic and anti-inflammatory therapy was repeated.

### Cartilage analysis

The cartilage samples collected during arthroscopy were cut in three fragments, used for: (1) histology; (2) immunohistochemistry for type II collagen; (3) metabolic labelling and analysis of proteoglycans (PGs).

### Histology

For histology, the tissue fragments were fixed in Methacarn (60% methanol, 30% chloroform, 10% acetic acid) for 6 h, and then transferred to 90% ethanol. The tissues were dehydrated, embedded in paraffin, and cut in 3 μm sections. These sections were transferred to silane-coated microscope slides, dewaxed and stained with hematoxylin and eosin (H & E).

### Immunofluorescence of type II collagen

For immunofluorescence labelling, the tissue fragments were fixed as above described, dehydrated, embedded in paraffin, and cut in 5 μm sections. These sections were transferred to silane-coated microscope slides and dewaxed. Antigen retrieval was performed by incubation with 0.4% pepsin (Sigma) in 0.5 M acetic acid, 30 min, 37°C. After washing with phosphate buffering saline (PBS), polyclonal anti-type II collagen antibody^c^ was added as primary antibody (100 μL), 1:50 in blocking solution containing 1% bovine serum albumin, BSA^d^, 0.3% Tween 20 and 0.1% sodium azide. This antibody binds both type II mature collagen and procollagen. After overnight incubation at 4°C, the slides were washed with PBS and incubated with secondary antibody swine anti-rabbit IgG, FITC-conjugated^e^, diluted 1:100 in PBS. After 90 min incubation in moist and dark chamber, the sections were counterstained with propidium iodide (1:1000), which stains cell nucleus in red. Slides were mounted in Vectashield to preserve fluorescence^f^, and sealed with clear nail polish. Negative controls consisted of cartilage sections that had not been incubated with primary antibody.

Images were obtained with a Nikon E-800 fluorescence microscope, 400 times magnification, and morphometric analysis was performed using the software Image ProPlus 4.5. The evaluation consisted of counting 100 cells per sample, in random selected fields, following basic stereological principles. The results are expressed in percentage of type II collagen -positive cells.

### Metabolic labelling and analysis of proteoglycans

For metabolic labelling of PGs, the cartilage fragment (~100 mg) was transferred to a sterile bottle containing 10 mL of F12 culture medium containing penicillin (10.000 U), streptomycin (100 mg) and ^35^S-sulfate^g^ (100 μCi). The tissue explants were transferred to Dept. of Biochemistry, Escola Paulista de Medicina, and maintained at 37°C in a 2.5% CO_2_ atmosphere. After 24 h, the medium and the tissue explants were collected and processed separately.

### Extraction of ^35^S-PGs from tissue explants

From the conditioned culture medium, the PGs were precipitated by careful and slow addition of methanol (3 volumes), and after 18 h at −20°C, the precipitate formed was collected by centrifugation, dried and resuspended in 100 μL of water for analysis.

From the tissue explants, ^35^S-PG extraction was performed as previously described [23]-[25]. Each tissue explant was carefully cut in small pieces, and incubated with 1 mL of 4 M guanidine hydrochloride (GuHCl)^h^ in 0.05 M sodium acetate buffer, pH 6.5, containing protease inhibitors (0.1 M є-aminocaproic acid, 6.5 mM benzamidine, 5.5 mM iodocetamide and 0.1 M phenylmethylsulfonyl fluoride). After overnight incubation at 4°C under agitation, debris was collected by centrifugation, and the extraction process was repeated with 0.5 mL of the 4 M GuHCl. After 24 h, debris was removed by centrifugation and both supernatants were combined. PGs were precipitated by slow addition of methanol (3 volumes), under agitation. Subsequently, the precipitates formed after 18 h at −20°C were collected by centrifugation (3000 × g, 20 min), washed with 80% methanol, and vacuum dried. The dried material was resuspended in water (100 μL), and the solutions were stored at −20°C.

### Identification and quantification of ^35^S-PGs

The ^35^S-PG samples were analyzed by a combination of agarose gel electrophoresis and enzymatic degradation with proteases and specific GAG lyases, as already described [26]. Aliquots of the ^35^S-PG-containing solutions (5 μl containing 0.5-5 μg) were submitted to agarose gel electrophoresis in 0.05 M 1,3-diaminopropane-acetate buffer (PDA), pH 9 [27]. After fixation with cetyltrimethylammonium bromide and Toluidine Blue staining, PGs were quantified by densitometry of the gel slabs^i^. The ^35^S-sulfate labelled compounds were visualized by exposure of the gel slabs to a Packard Cyclone TM Storage Phosphor System by 24 hours. For quantification, the bands containing radiolabelled compounds were scrapped from the gel slabs and counted in a liquid scintillation spectrophotometer, using Ultima Gold LSC-Cocktail^j^. The quantitative results were always corrected for ^35^S-decay.

### Statistical analysis

Data were evaluated for normality by the Kolmogorov-Smirnov test. Afterwards, the Analysis of Variance was used to compare the groups and effect of treatments. To contrast between mean values the Tukey-Kramer test was used. For non-parametric values, Kruskal-Wallis test was used to compare initial and final results, while Mann–Whitney test was used to compare the chitosan-GP-treated joints to the untreated. The GraphPad Instat 3 was used to perform the statistical analysis.

## Results

### Histology and immunohistochemistry of type II collagen with fluorescence labelling

**Figure 1:**
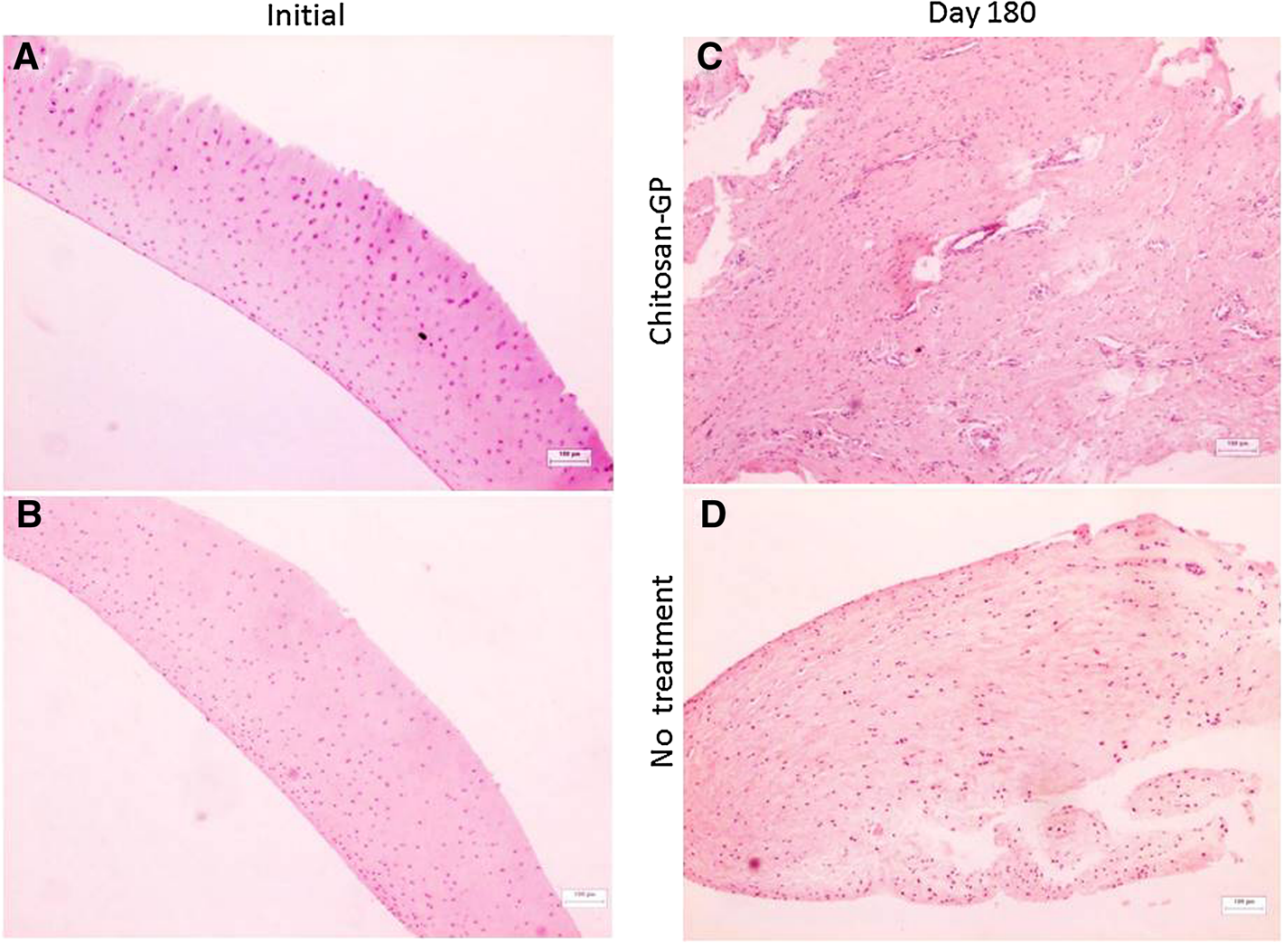
**Optical microscopy of normal articular cartilage (A and B, Initial) collected at the first arthroscopy to produce the osteochondral defect, and the healing tissues (Final, day 180) formed in presence (C) or absence of chitosan-GP (D).** Tissue samples collected during surgery were fixed in Methacarn, transferred to 90% ethanol, dehydrated, embedded in paraffin and cut in 3 μm sections. These sections were transferred to silane-coated slides, dewaxed, and stained with hematoxylin and eosin (H & E).

Gross observation revealed that at the beginning of experiment, the articular cartilage was normal with smooth surface. At the end of experimental period (day 180), a slight depression was easily detected by arthroscopy as the lesion was observed. The surface was also smooth and cartilage-like. Histological images of the initial chondral fragment obtained by arthroscopy is shown in Figure 1 (A and B). Chondrocytes are embedded in abundant avascular ECM of homogeneous appearance upon HE staining. Figure 1-C shows that, 180 days after osteochondral injury (Final), the chitosan-GP treated tissue had an abnormal, disorganized appearance. The cells were also embedded in abundant ECM, but now blood vessels were present. In both healing tissues, some cells presented a “fibroblastic” morphology. Possibly, they are mesenchymal cells that could differenciate into either chondrocytes or fibroblasts. The total number of cells per field did not change, and macrophages and polymorphonuclear cells were not detected, suggesting that no significant inflammation was present in the “Final” samples (180 days). Similar results were obtained for all animals, and Figure 1 shows representative samples of each group.

**Figure 2:**
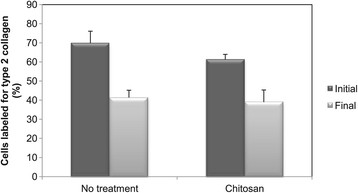
**Percentage of cells stained for type II collagen in normal articular cartilage (Initial) and in healing tissues formed in presence or absence of chitosan-GP.** Cells were counted under fluorescence microscope, and the relative number of type II collagen-positive cells (green and yellow cells) is given (as percent). Brackets indicate differences statistically significant (*P* < 0.05).

Immunofluorescence for type II collagen was performed as described in Methods, and the percentage of type II collagen-positive cells was measured (Figure 2). Although the matrix was also labelled, our focus was the newly synthesized pro-collagen present in the cells. In normal cartilage, about 70% of the cartilage cells were type II collagen-positive, while in healing tissues this number was much lower (~40%). Nevertheless, there was no difference between healing tissues formed in presence or absence of chitosan-GP.

### Proteoglycans

**Figure 3:**
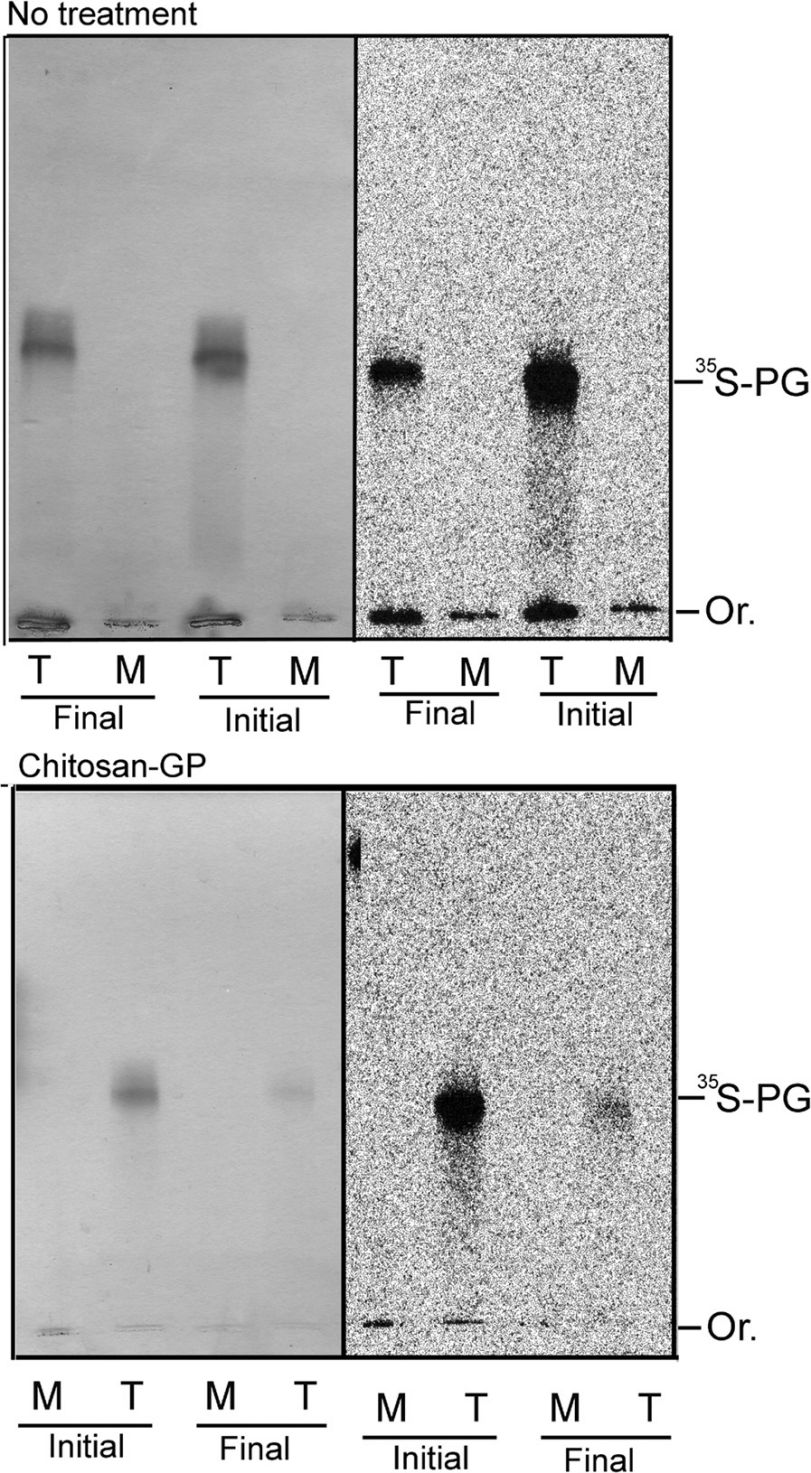
**Agarose gel electrophoresis of**^**35**^**S-PGs from normal articular cartilage (Initial) and healing tissue (Final, 180 days) formed either in absence (No treatment) or in presence of chitosan-GP.** Normal articular cartilage samples collected during the first arthroscopy to produce the osteochondral defect (~100 mg, wet weight), as well as samples of the healing tissues formed in presence or absence of chitosan-GP, were metabolically labeled with ^35^S-sulfate. ^35^S-PGs were isolated from the conditioned media (M) and the tissue explants (T). These compounds were analyzed by agarose gel electrophoresis (PDA buffer), and localized in the gel by Toluidine Blue staining (left) and radioautography (right).

Representative agarose gel electrophoresis of ^35^S-PG extracted from cartilage explants (T) and conditioned culture medium (M), from normal tissues obtained at the beginning of experiment (Initial), and repair tissues collected at the end of experimental period (Final), from chitosan-GP-treated lesions (Chitosan-GP) and untreated lesions (No treatment) is shown in Figure 3. Both Toluidine Blue staining and radioautograms are shown. The radioactive band corresponded to the Toluidine Blue stained PGs, indicating that PGs were the main ^35^S-sulfate labelled compounds. Intact PGs migrated near heparan sulfate (free GAG), but upon digestion with proteases (not shown), only chondroitin sulfate free chains appeared, indication that most of the ^35^S-PGs synthesized are chondroitin sulfate-PGs.

**Figure 4:**
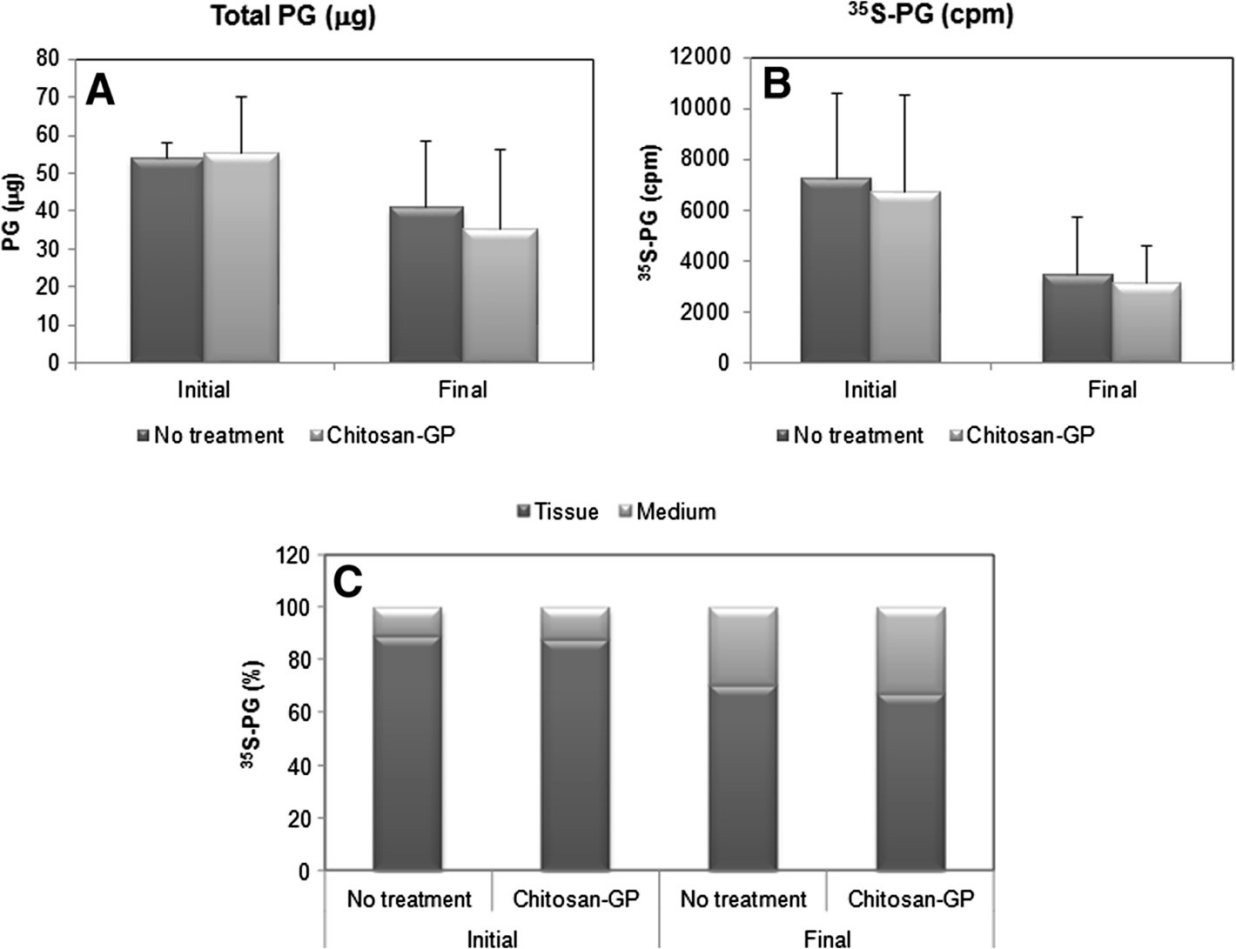
**Total and**^**35**^**S-PGs from normal articular cartilage (Initial) and healing tissue (Final) formed in presence or absence of chitosan-GP.** The experiment was performed as described in Figure 4, except that quantitative data are shown for total PGs **(A)**, quantified by densitometry of the Toluidine Blue-stained agarose gel electrophoresis, and ^35^S-PGs, quantified by the radioactivity of the ^35^S- containing bands **(B)**. Mean ± standard error is shown in **(A)** and **(B)**, while **(C)** shows the distribution of ^35^S-PGs in culture medium and tissue explants.

Figure 4 shows the quantitative data for total PGs (stained by Toluidine Blue and quantified by densitometry) and newly synthesized, labelled PGs (^35^S-PGs). In all cases, most of the ^35^S-PGs were found in the tissue explants, with only 10-12% of total ^35^S-PGs in culture medium of normal tissues (Initial). Although both the total amounts of PGs (Figure 4-A) and ^35^S-PGs (Figure 4-B) were lower in healing tissues, a higher proportion (30-33%) was found in culture media (Figure 4-C), suggesting that the extracellular matrix was less dense than in the normal tissue. The healing tissues contained also lower amounts of PGs, detected both by Toluidine Blue staining and radioactivity.

**Figure 5:**
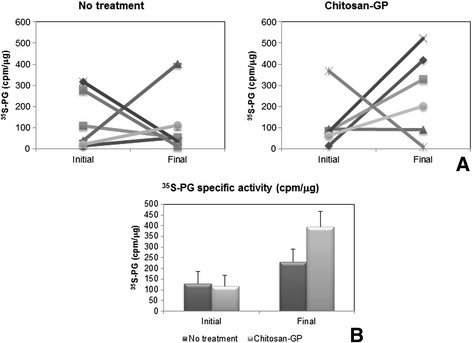
**Individual and mean**^**35**^**S-PGs specific activities from normal articular cartilage (Initial) and healing tissue (Final) formed in presence or absence of chitosan-GP.** The experiment was performed as described in Figure 3, except that only the PGs extracted from tissue explants were considered. Specific activity is expressed as cpm/μg of tissue protein. Individual paired results, and mean ± standard error are shown in **(A)** and **(B)**, respectively.

The PG concentration in the culture medium was very low, undetectable by Toluidine Blue staining. For this reason, it was impossible to determine the specific activities of PGs secreted to the culture medium. So, only tissue explants were considered to measure specific activities. In healing tissues, although the amounts of PGs were lower than normal, the specific activities were increased (Figure 5), indicating higher synthesis rates. The specific activities were even higher in the chitosan-GP-treated lesions. Nevertheless, individual variation did occur, possibly due to different numbers of PG-synthesizing cells in each sample.

## Discussion

Much of the potential of chitosan as a biomaterial is thought to come from its cationic nature and high charge density. Because the chitosan charge density is pH-dependent, transfer of chitosan-polyanion complexes to physiological pH can result in dissociation of the immobilized polyanion, and this could be used for local delivery of biologically active molecules [28]. Furthermore, the N-acetylglucosamine moiety of chitosan is also found in some GAGs, such as hyaluronic acid, heparan suphate and heparin, giving these molecules a certain degree of structural analogy. It is possible that chitosan might present some of the GAG biological activities, such as the ability to bind growth factors, receptors and adhesion molecules. However, Hamilton et al. claim that other properties may be important in biological performance of chitosan [29].

Many authors propose chitosan-based biomaterials for cartilage tissue regeneration, but different chitosan formulations are used. The chitosan molecular weight may range from as little as 10 kDa to over 1,000 kDa, while the deacetylation degree may oscillate from 50% to 95% [30]. High molecular weight and high deacetylation degrees are related to lower material degradation, while the deacetylation degree is also related with cell adhesion and proliferation. In the present paper, we choose to employ an intermediate molecular weight, and a 310–375 kDa chitosan, with 75% deacetylation was used.

The chitosan primary amino groups are reactive and provide a mechanism for side group attachment using mild reaction conditions. The general effect of addition of a side group is to disrupt the crystalline structure of chitosan and hence increase the amorphous fraction, generating a material with lower stiffness and altered solubility. Chitosan-GP is one such compound [19], having the additional benefit of forming a thermosensitive hydrogel that forms a viscous liquid at room temperature, and converts to a semisolid gel at body temperature, making easier its application in osteochondral defects. There are strong evidences that the gelation in the chitosan-GP system induced by heating is the result of a progressive and homogeneous reduction of the ionization degree of chitosan [31].

Other carbohydrate-based biomaterials, such as polylactic/polyglycolic acids, agarose, and alginate, have been used, but most of them, in contrast to chitosan, give strong foreign body reactions review in [32].

Although thoroughly studied in humans and in experimental models reviews in [1],[14], only a few reports on the use of chitosan-based biomaterials in equines have been published [33], and while the body response to various chitosan-based implants has been studied in other species [30],[34], there are scarce studies in horses. In general, studies in other species have revealed that chitosan-based materials are biocompatible and evoke minimal foreign body reaction. Chitosan, chitosan fragments, and chitin have been shown to have short-term stimulatory effects on the immune system [35]-[37], which may play a role in inducing local cell proliferation and ultimately integration of the implanted material within the host tissues. In the rabbit, it was shown that less adverse tissue response occurred with better biodegradable samples, of low molecular weight chitosan and low deacetylation degree (83%) [30]. With high molecular weight chitosan (350–510 kDa) or high deacetylation degree (91%), however, Abarrategi et al. described synovial membrane inflammation, no cartilage formation, no material degradation, and nonsmooth surface [30]. In contrast, our findings in horses, using chitosan-GP of 310–375 kDa and 75% deacetylation, revealed smooth surface, with a good host integration of healing tissue, and no evidence of chronic inflammation in any of our experimental animals. Blood vessels were observed in repair tissues formed in presence of chitosan-GP, as were also reported by others, who concluded that chitosan induces a transient vascularization when implanted in cartilage [30]. In a rabbit model, chitosan-GP/blood implants induce angiogenesis and subchondral bone remodeling. These observations suggest that chitosan-GP/blood implants leads to a porous subchondral bone structure with well vascularized marrow cavities, that could promote more effective cartilage repair [38].

Histology of the repair tissue did not show any changes in the number of cells per field, and macrophages and polymorphonuclear cells were not detected, indicating that the implanted chitosan-GP did not evoke an important inflammatory reaction, while permitting cell proliferation. These cells were able to synthesize type II collagen and PGs, suggesting its “chondrocyte” nature. Nevertheless, the cell population in healing tissues, both in presence of chitosan-GP and in untreated controls, were heterogeneous: only ~40% of the cells were type II collagen-positives and some presented a fibroblastic aspect under light microscopy. A histological scoring system, as well as staining with Safranin-O, Toluidine Blue, and use of polarized microscopy for collagen could be useful to further characterize the healing tissues. However, tissue fragments were differently processed for histology, immunofluorescence, and proteoglycan synthesis under tissue culture conditions, leaving not enough material left for the above mentioned analyses.

Moreover, the PGs synthesized in presence of chitosan-GP were similar to those synthesized in healing tissue without implant, and also to normal cartilage, except that higher proportions of PGs were found in the culture medium of repair tissues. This suggests that the extracellular matrix of the newly synthesized tissue is less dense, in agreement with the findings on type II collagen labelling (see Figures 2 and 4). Chitosan-GP in horses did not increase the PG synthesis rate, although the specific activity of ^35^S-PGs was slightly increased (difference not statistically significant, Figure 5). This is also in agreement with previously reported data on chondrocytes cultured on chitosan-chondroitin sulphate complexes [14], which produced equal to control amounts of PGs and type II collagen. Nevertheless, the specific activity of ^35^S-PGs varied among different animals. This is possibly due to the variations in the number of PG-synthesizing cells in each sample.

It is worth mentioning that these results are related to the osteochondral defect model here employed. In the equine species, the relevance of this model resides in the high prevalence of osteochondral lesions, both acquired or inherited [39],[40]. Furthermore, the choice of the depth of the articular cartilage defect has great impact in the tissue response. Osteochondral defects, as employed in the present study, span the entire depth of articular cartilage and penetrate subchondral bone marrow, creating an access to blood cells, macrophages, mesenchymal cells and soluble factors [41]. Osteochondral defects exhibited a rapid cellular response, as opposed to chondral defects, which exhibited a minor healing response, with little cell infiltrates [42]. It is also important to mention that both models of defect design – chondral and osteochondral – are relevant for equines [43],[44] as well as for other species [32],[41], and the choice to use one or the other must be made judiciously.

## Conclusions

Despite extensive research on articular cartilage healing during the last decades, successful repair of damaged cartilage has not been fully achieved. While many new techniques have recently emerged and demonstrated promising results in experimental models, few have exhibited long-term clinical efficacy. Tissue engineering has recently emerged as an interdisciplinary topic, using *in vitro* cultured cells and tissues, together with artificial implants. Our findings on chitosan-GP suggest that it may be well suitable as a biomaterial for cartilage repair [45] that could also be used in drug and/or cell delivery [46]. Future studies using *in vitro* cultured chondrocytes will explore this potential.

## Endnotes

^a^Cat #9012-76-4, 419419, Sigma-Aldrich, MO, USA;

^b^Cat #55076-41-1, Sigma-Aldrich, MO, USA;

^c^Cat# 600-401-104-0, Rockland, USA;

^d^Cat# A9647, Sigma, MO, USA; 

^e^Dako, Carpinteria, CA, USA;

^f^Vector Laboratories, Inc, Burlingame, CA, USA;

^g^IPEN-CNEM, São Paulo, SP, Brazil;

^h^Sigma-Aldrich Chemical Co. Inc, Milwaukee, WI, USA;

^i^Scanner CS-9000, Shimadzu; 

^j^Packard Instruments Company Inc., Downers Grove, IL, USA.

## Abbreviations

ECM: Extracellular matrix

PGs: Proteoglycans

GAGs: Glycosaminoglycans

GP: Glycerol phosphate

H & E: Hematoxylin and eosin

PBS: Phosphate buffering saline

GuHCL: Guanidine hydrochloride

PDA: 1,3-diaminopropane-acetate

## Competing interests

The authors declare that they have no competing interests.

## Authors’ contributions

LCLCS designed the study and was responsible for obtaining funds. LCLCS and EANM performed all the arthroscopies. EANM and RYAB collected tissue samples and conducted the experimental analysis. RYAB performed the metabolic labelling, isolation, and characterization of proteoglycans, under the supervision of YMM, and participated in the writing and revision of the manuscript. YMM designed and supervised the studies on cartilage proteoglycans, and wrote the manuscript. BC performed the histological analysis. All authors read and approved the final manuscript.
